# Development of a Liquid Chromatography and High-Resolution and -Accuracy Mass Spectrometry Method to Evaluate New Biotherapeutic Entity Processing in Human Liver Lysosomes

**DOI:** 10.4049/immunohorizons.2300035

**Published:** 2023-06-16

**Authors:** Gabriele Sergio Colangelo, Andrea Di Ianni, Kyra Cowan, Federico Riccardi Sirtori, Luca Maria Barbero

**Affiliations:** * University of Turin, Molecular Biotechnology Center, Turin, Italy; † NBE-DMPK Innovative BioAnalytics, RBM Merck S.p.A., an affiliate of Merck KGaA, Darmstadt, Germany, Colleretto Giacosa, Torino, Italy; ‡ New Biological Entities, Drug Metabolism and Pharmacokinetics, Research and Development, Merck KGaA, Darmstadt, Germany

## Abstract

Biotherapeutic immunogenicity remains a great challenge for researchers because multiple factors trigger immune responses. Predicting and assessing the potential human immune response against biological drugs could represent an impressive breakthrough toward generating potentially safer and more efficacious therapeutic proteins. This article describes an in vitro assay that can contribute to evaluating the potential immunogenicity of biotherapeutics by focusing on lysosomal proteolysis. We selected human liver lysosomes (hLLs) from four different donors as a surrogate in vitro model instead of APC lysosomes because they are a ready-to-use lysosomal source. To assess the biological comparability of this surrogate to APC lysosomal extract, we compared the proteome content of hLLs with literature data of lysosomal fractions extracted from murine bone marrow and human blood-derived dendritic cells. Then we tested infliximab (IFX; Remicade) under different proteolytic conditions using liquid chromatography and high-resolution and -accuracy mass spectrometry to better define the degradation kinetics inside the lysosomes. hLLs revealed similar enzymatic content compared with human and murine dendritic cell lysosomes. Degradation assays demonstrated that our liquid chromatography and high-resolution and -accuracy mass spectrometry method could identify both the intact protein and the peptides resulting from proteolysis with high specificity and resolution. The rapid and easy assay described in this article can be extremely useful for evaluating the immunogenic risk associated with therapeutic proteins. In addition, this method can complement information from MHC class II–associated peptide proteomics assays and other in vitro and in silico techniques.

## Introduction

Therapeutic proteins developed for complex and severe diseases play a crucial role in modern medicine (1). However, they can show some critical issues when it comes to evaluating their safety and efficacy in in vivo systems. Among the major concerns to tackle, the potential immunogenicity stands out as one of the main causes of drug failure in the molecule design phase. The importance of assessing the immunogenicity risk of novel developing biotherapeutics has been recognized by regulatory authorities and industry, leading to the onset of derisking strategies based on in silico predictions and in vitro/ex vivo assays (2, 3). Because therapeutic proteins might be deemed as non–self-antigens by the human immune system, they may trigger adverse immune responses that can be a direct cause of safety issues for patients. Immunogenicity of these biotherapeutics can also compromise their pharmacokinetics, and pharmacodynamics through the generation of antidrug Abs (ADAs), which can jeopardize the success of the clinical outcomes and, in the worst case, lead to drug disapproval or withdrawal (4–8). For this reason, having derisking strategies in place before final molecule design can mitigate the immunogenicity risk of novel biotherapeutics.

APCs, such as dendritic cells (DCs), macrophages, and activated B cells, trigger CD4^+^ T cells by presenting immunogenic peptides of an Ag on the MHC class II (MHCII) receptor (9). Peptides presented by MHCII molecules are derived from proteins that have been cleaved by specific processes within the endosomal compartments, providing a way for CD4^+^ T cells to respond to exogenous Ags internalized by APCs through several mechanisms (10–12). In endolysosomal compartments, proteins with stiff native conformations as a result of disulfide bridges can undergo reductive processes that contribute to the exposure of the protein sequence to lysosomal enzymes (13–15). It implies that such proteases have easier access to the Ag backbone, which is more cleavable into smaller peptides (16). The newly formed peptide clusters are then loaded on the MHCII binding groove trafficked through the cytosol to the cell membrane (12). Because peptide–MHCII is recognized by CD4^+^ T cells, these cells become activated, differentiate into Th cells, and mediate interactions between Ag-specific B cells and Th cells.

The immunogenicity of Ags highly depends on the character of their interaction with DCs. However, other factors can contribute to immunogenic Ag properties, such as structural features, aggregation, posttranslational modifications, and stability, which might affect the internalization of biotherapeutics and their processing efficacy and site specificity, contributing to their immunogenic properties (17–22). According to the results of Lam et al. (23), some glycosylated proteins cause a more intense proliferation of T cells than their nonglycosylated counterparts, possibly because of differences in the internalization of those proteins. Moreover, structural variations between isoforms can lead to differences in terms of internalization and endocytosis, causing a slower and less efficient T cell activation, as described by Egger et al. (24) in their work on both variants of birch pollen allergen. It has also been demonstrated that the conformational stability of an Ag controls protein processing and presentation by APCs. When proteins are subjected to physical or chemical stresses, such as pH, temperature, or redox environments, they experience unfolding processes, allowing proteases better access to the peptide backbone (25, 26). Hence the intrinsic stability of Ags affects the kinetics of proteolysis degradation, thereby determining the availability of appropriate Ag peptides for MHCII loading and, ultimately, immunogenicity and T-cell polarization (27, 28). In this regard, disulfide bridges can reinforce and stabilize proteins in a well-defined conformation. Therefore, epitopes that are not normally cleaved and exposed on the cell surface could be expressed after the reduction of disulfide bridges. This implies a change in the composition of the immunopeptidome displayed on the cell surface and, therefore, a different CD4^+^ Th cell response (29). In this context, IFN-γ–induced thiol-reductase enzyme (GILT) is described to contribute to the reducing activity of the MHCII compartments, thus reducing disulfide bridges and consequently facilitating the unfolding of proteins and their subsequent cleavage (30–34).

**FIGURE 1.**
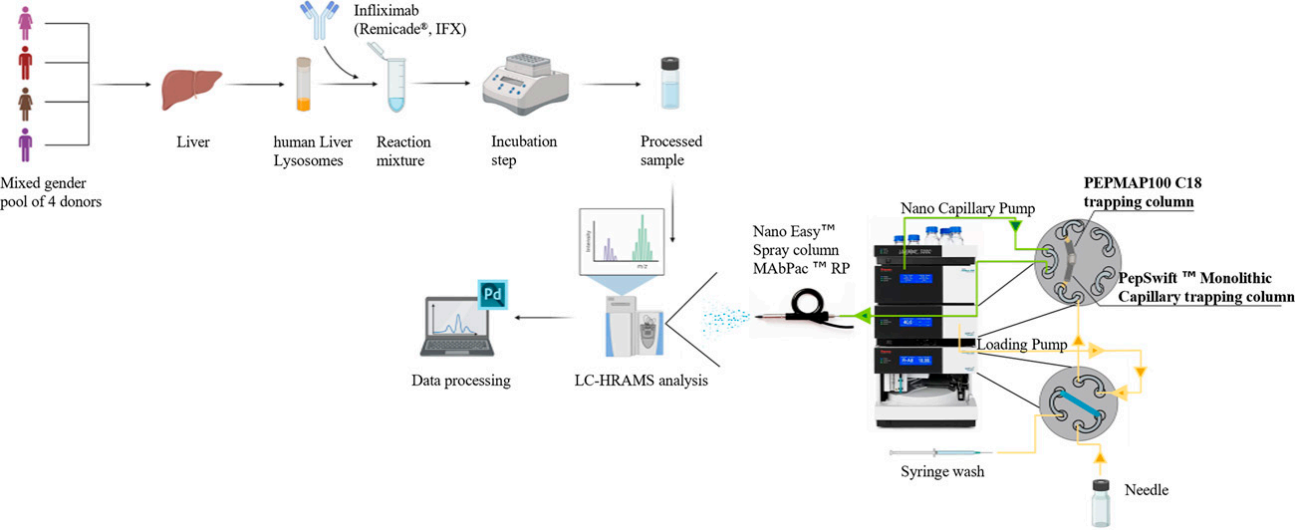
Schematic representation of rationale used for the protein-processing analysis using hLLs. Lysosomal extracts from a mixed gender pool of four donors were used as biological substrate for assessing the biotherapeutics degradation. Intact mass degradation and relative cleaved peptides were analyzed by performing a single analytical run through a nano–liquid chromatography system coupled to a high-resolution MS. Two pretrapping chromatographic columns with different stationary phases were serially connected to pretreat the sample before the analytical separation. MS2 data were elaborated by PD. The figure was created with BioRender.com.

In this article, we report a specific liquid chromatography and high-resolution and -accuracy mass spectrometry (HRAMS; LC-HRAMS) method to better understand the Ag proteolysis in lysosomes focusing on GILT activity in different redox conditions using several reducing agents. The anti–TNF-α mouse-human IgG1/κ Ab (infliximab [IFX] [Remicade]) was tested as immunogenic protein using a pool of human liver lysosomes (hLLs) from four different donors as a high-quality, easy, and ready-to-use in vitro system. IFX in vitro immunogenicity has already been described in the literature (35). Moreover, clinical studies confirmed that patients affected by rheumatoid arthritis and other inflammatory diseases developed ADAs against IFX, resulting in a compromised therapeutic outcome (36, 37). Considering the molecular complexity of the samples, we developed an LC-HRAMS method that can identify the large number of fragments originating from the lysosomal processing and the test molecule in the same analytical run. In short, this multimethod gathers bottom-up and top-down analysis approaches. As such, cleaved peptides were identified by tandem mass spectrometry (MS), whereas intact protein degradation was analyzed by the intact mass full-scan MS (Fig. 1). IFX degradation rate was evaluated, and the identified peptide clusters were matched to data from in-house–performed MHC class II–associated peptide proteomics (MAPPs) assay where IFX was tested using monocyte-derived DCs (mDCs) from several healthy donors.

## Materials and Methods

### Chemicals

All solvents and reagents used to perform the experiments were commercially available (Sigma-Aldrich–Merck Life Sciences, St. Louis, MO), and no additional purification steps were necessary. DTT and cysteine (Cys) were purchased from Roche Diagnostic (Mannheim, Germany) and Sigma-Aldrich–Merck Life Sciences, respectively. IFX was synthetized by Creative Biolabs laboratories (Shirley, NY). All the reagents and kits used for SDS-PAGE separations and protein digestion were purchased from Thermo Fischer Scientific (Waltham, MA).

### Human liver lysosomal extracts from a mixed gender pool of donors

Human liver lysosomal extracts (Lot No. 2010245), from a mixed gender pool of four donors, were purchased from Sekisui Xenotech (Kansas City, KS) at a concentration of 2.0 mg protein/ml in a suspension medium of 250 mM sucrose and 10 mM HEPES at pH 7.0. In brief, intact lysosomes were isolated from nontransplantable, fresh, whole-liver tissues by multiple density gradients and ultracentrifugation steps, as described elsewhere (38). The tissues were cooled and flushed with either University of Wisconsin or histidine tryptophan ketoglutarate cold preservation solution and packed on ice immediately after the transplant. Then they were treated with a PBS-based buffer solution to flush out the cold preservation solution and any remaining blood cells that might be still present after the transplant. After the lysosomes were isolated, they were homogenized by sonication for ease of use and experimental reproducibility.

### Human liver lysosomal fingerprinting

A total of 20 µl of lysosomal proteins (2 mg/ml) was digested using Thermo Scientific SMART Digest Kit (Thermo Fisher Scientific). A total of 150 µl of Thermo Scientific SMART Digest Buffer, 30 µl of ultrapure water, 20 µl of hLLs, and nonmagnetic trypsin-coated beads (one vial) were added into a 2-ml polypropylene low binding protein Eppendorf tube (2-ml LoBind tube Safe Lock tubes PCR clean; Eppendorf SE, Hamburg, Germany). The solution was digested on a thermomixer at 70°C/1400 rpm for 1 h. At the end of the digestion, the tube was transferred into a bench centrifuge at 13,000 × g for 10 min at 4°C. A total of 100 µl of sample supernatant was taken and transferred into a new low binding protein Eppendorf tube and then diluted 1:1 with 100 µl of HCOOH 2%. The solution generated (0.1 mg/ml) was centrifuged at 17,000 × g for 5 min at 4°C, and 30 µl of supernatant was withdrawn and moved into a new autosampler vial (QuanRecovery MaxPeak tubes, polypropylene 300 µl; Waters, Milford, MA). The remaining solution was immediately stored at −80°C. A total of 1 µl of trypsinized lysosomal proteins was injected into a nanoflow ultra-performance liquid chromatography system (Thermo Scientific UltiMate 3000 RSLC nano system; Thermo Fisher Scientific) using a 90-min gradient with a loading pump starting flow rate of 0.010 ml/min (solvent B: acetonitrile (ACN)/0.1% HCOOH; solvent C: ACN in 0.05% aqueous trifluoroacetic acid ; 0–4 min 100% C at 0.010 ml/min, 4–5 min 95% B/5% C at 0.050 ml/min, 5–20 min 95% B/5% C at 0.050 ml/min, 20–21 min 100% C at 0.050 ml/min, 21–36 min 100% C at 0.050 ml/min, 36–37 min 100% C at 0.010 ml/min, 37–90 min 100% C at 0.010 ml/min), and a Nano Capillary pump flow rate of 0.400 µl/min (solvent A: 0.1% HCOOH; solvent B: 0.1% HCOOH/ACN; 0–4 min 2% B at 0.400 µl/min, 4–54 min 40% B at 0.400 µl/min, 54–59 min 95% B at 0.400 µl/min, 59–69 min 95% B at 0.400 µl/min, 69–71 min 2% B at 0.400 µl/min, 71–90 min 2% B at 0.400 µl/min). The trapping and the analytical column used for the separation were PEPMAP100 C18 5 µm 0.3 × 5 mm 5PK (Product No: 160454; Thermo Fisher Scientific) and MAbPac RP UHPLC Column 4 µm, 1500 Å, 150 µm × 15 mm (Product No. ES907; Thermo Fisher Scientific), respectively. The analytical column was kept at the temperature of 35°C for the entire chromatographic run, whereas the trapping one was at room temperature. The chromatographic setup was coupled to a Q Exactive Plus Orbitrap Mass Spectrometer (Thermo Fisher Scientific), and spectra were generated using a nano electrospray (ESI) ionization source (EASY-Spray Source; Thermo Fisher Scientific) in ion-positive mode. Transition from full mass to data-dependent acquisition (DDA) was used, with full MS1 scans measured in the mass range from 266.7 to 4000 m/z up to 70 min, at a resolution of 70,000 full width at half maximum (FWHM) at 200 m/z with a maximum injection time of 100 ms and automatic gain control (AGC) target of 3 × 10^6^. Fragmentation was achieved using high-collision energy dissociation. The isolation window was set to 2.0 m/z without isolation offset. The resolution in MS2 was at 17,000 FWHM with a maximum injection time of 100 ms and AGC target of 1 × 10^5^. Full mass acquisition was used in a mass range from 333.4 to 5000 m/z from 70 to 90 min, at a resolution of 17,500 FWHM at 200 m/z with a maximum injection time of 200 ms and AGC target of 3 × 10^6^. S-lens radiofrequency levels were set to 80 Hz for the intact detection and 50 Hz for the peptide analysis, respectively. A tandem MS database search program (Sequest search engine) and the software Proteome Discoverer 2.5.0.400 (PD; Thermo Scientific) were used for peptide identification with the following parameters: taxonomy, homo sapiens; variable modification, methionine and tryptophan oxidation, asparagine, glutamine, and arginine deamidation; enzyme, Trypsin (full); precursor ion mass accuracy, ±10 ppm; product ion mass accuracy, ±0.02 Da; maximum missed cleavages, 2; false discovery rate, 1%.

### IFX degradation assay

IFX Remicade (150.085 µg/ml, 1.01 µM), catabolic buffer 10× containing 2 mM DTT (product no. K5200; Sekisui Xenotech, Kansas City, KS), and HLLs (0.125 mg/ml) (Lot No. 2010245; Sekisui Xenotech) were diluted in liquid chromatography MS–grade water up to 420 µl and added into a 2-ml polypropylene low binding protein Eppendorf tube. Moreover, DTT (500 mM) and Cys (100 mM) were used as additional reducing agents to obtain the desired redox conditions. Three stock solutions were generated and stirred on a thermomixer at 37°C/1100 rpm up to 72 h, and an aliquot of 50 µl was collected at each time point. Samples were collected at the following time points: 0, 0.5, 1.5, 3, 5, 24, 48, and 72 h; and they were flash frozen at −80°C. Before injecting samples in the HPLC system, they were thawed and then centrifuged at 13,000 × g for 10 min at 4°C. A total of 30 µl of supernatant was taken, diluted 1:1 with HCOOH 2%, and transferred into a new autosampler vial. Samples were analyzed in a UHPLC system (UltiMate 3000 RSLCnano system; Thermo Fisher Scientific) coupled to a Q Exactive Plus Orbitrap Mass Spectrometer, and spectra were generated in ESI (electrospray)-positive ion mode. Data-dependent acquisition was used for peptide detection, with full MS1 scans measured in the mass range from 266.7 to 4000 m/z up to 55 min at a resolution of 70,000 FWHM at 200 m/z with a maximum injection time of 100 ms and AGC target of 3 × 10^6^. Fragmentation was achieved by using high-collision energy dissociation. The isolation window was set to 2.0 m/z without isolation offset. The resolution was at 17,000 FWHM with a maximum of injection time of 100 ms and AGC target of 1 × 10^5^. For the intact protein detection, full mass acquisition was used in a mass range from 333.4 to 5000 m/z from 55 to 90 min, at a resolution of 17,500 FWHM with a maximum injection time of 200 ms and AGC target of 3 × 10^6^. The chromatographic separation was performed in two-dimensional mode, exploiting an analytical column and two trapping columns connected by a column union. The analytical column used was a MAbPac RP UHPLC Column 4 µm, 1500 Å, 150 µm × 15 mm (Product No. ES907), whereas the trapping columns were PEPMAP100 C18 5 µm 0.3 × 5 mm 5PK (Product No: 160454) and a PepSwift Monolithic Capillary UHPLC Column, 200 µm × 5 mm, Monolithic PS-DVB, respectively. Both intact proteins and cleaved peptides were eluted using a 90-min gradient with a loading pump starting flow rate of 0.015 ml/min (solvent B: ACN/0.1% HCOOH; solvent C: ACN in 0.05% aqueous trifluoroacetic acid; 0–15 min 2% B at 0.015 ml/min, 15–16 min 95% B at 0.050 ml/min, 16–27 min 95% B at 0.050 ml/min, 27–28 min 2% B at 0.050 ml/min, 28–38 min 2% B at 0.050 ml/min, 38–90 min 2% B at 0.015 ml/min) and a Nano Capillary pump flow rate of 1.000 µl/min (solvent A: 0.1% HCOOH/H2O; solvent B: 0.1% HCOOH/ACN; 0–4 min 2% B at 1.000 µl/min, 4–58 min 40% B at 1.000 µl/min, 58–60 min 80% B at 1.000 µl/min, 60–65 min 80% B at 1.000 µl/min, 65–65.100 min 95% B at 1.000 µl/min, 65.1–70 min 95% B at 1.000 µl/min, 70–70.1 min 2% B at 1.000 µl/min, 70.1–75 min 2% B at 1.000 µl/min, 75–75.1 min 80% B at 1.000 µl/min, 75.1–79 min 80% B at 1.000 µl/min, 79–79.1 min 2% B at 1.000 µl/min, 79.1–90 min 2% B at 1.000 µl/min). Peptide identification was performed using Sequest search engine against Swiss-Prot human database containing the sequence of the evaluated protein and PD (Thermo Scientific). The search has been performed with a mass tolerance of ±10 ppm for precursor ions and ±0.02 Da for fragment ions. Met-sulfoxide, Asn/Gln deamidation, and N-terminal pyroglutamylation have been considered as variable modifications. Data have been searched without enzyme specificity, and peptide results have been reported at 1% specific false discovery rate cutoff. Peptides showing >1.9 or >2.3 of cross-correlation value for doubly or triply charged ions, respectively, and <0.1 of the δ cross-correlation have been considered as true hits. Only 9–30 mer peptides were considered, according to our purposes.

### SDS-PAGE electrophoresis

Samples were resolved on SDS-PAGE 10% precast polyacrylamide mini gels (NuPAGE 10%, Bis-Tris, 1.0–1.5 mm; Invitrogen, Thermo Scientific). Untreated and treated samples were reduced by using NuPAGE Sample Reducing Agent (10×) (Invitrogen, Thermo Scientific), and Pierce LDS Sample Loading Buffer (4×) (Invitrogen, Thermo Scientific) was added to make proteins unfold in the presence of lithium dodecyl sulfate. Both reagents were diluted to 1× final concentration. Samples were heated up on a preheated thermomixer at 70°C/1100 rpm for 5 min and then pipetted into a 10% precast mini gel wells. A total of 1.5 µg of treated IFX and 4 µg of untreated IFX were loaded in wells. 20× Bolt MES SDS Running Buffer (Invitrogen, Thermo Scientific) was used at a final 1× concentration as running buffer, and 400 µl of Bolt Antioxidant (Invitrogen, Thermo Scientific) was added in to avoid sample reoxidation and maintain proteins in a reduced state during protein gel electrophoresis and protein transfer. The electrophoretic run was performed at a constant voltage of 200 V for 30 min. A ready-to-use Coomassie G-250 stain (SimplyBlue SafeStain; Invitrogen, Thermo Scientific) was used for 2 h at 37°C for visualizing protein bands on polyacrylamide gel.

### Statistical analysis

Kinetic parameters related to IFX degradation in matrix considered were calculated by Prism 4.03 (GraphPad Software) for each redox condition. Three independent replicates were performed to assess IFX catabolism in terms of biological variability. A label-free quantification (LFQ) approach was carried out to evaluate statistical differences among experimental conditions by using PD (Thermo Scientific). Peptide abundances were calculated considering the 24-h time point as a reference point, because at this time point, the highest number of peptides and proteins was found. Such analyses were performed for each redox condition tested. The number of common peptides among biological replicates was calculated and displayed by Euler-Venn graphs.

## Results

### Human DC and liver lysosomal fingerprinting displayed high enzymatic compatibility

Human liver lysosomal fingerprinting was carried out by using a shotgun proteomic in vitro approach exploiting thermostable trypsin-coated beads to cleave proteins into smaller peptide chains. Subsequently, the peptide pool was analyzed through HRAMS. More than 2800 proteins were identified in hLLs. Proteins related to the endolysosomal proteolytic machinery or involved in the lysosomal processing were listed and compared with those identified in mDCs and bone marrow-derived dendritic cells (BMDCs) by Egger and coworkers (24) (Table I). Most of the detected enzymes belonged to the large family of cathepsins and were involved in protein digestion inside lysosomal compartments. Trypsin-digested protein % coverage of cathepsin D, L, S, B, H, and Z levels was slightly lower, although still comparable with those reported in the identification in DC-derived lysosomes. Cathepsin K, F, and G were unexpectedly found only in hLLs; on the contrary, the isoforms A and C were not revealed. The percentages of protein coverages for asparagine endopeptidase endoprotease (legumain), dipeptidyl peptidase 2, and tripeptidyl peptidase 1 exoproteases were similar between hLLs and BMDCs. Other enzymes related to lysosomal processing, although not strictly involved in the peptide backbone fragmentation, were also identified. In this regard, the MS analysis confirmed the presence of GILT in the human lysosomal extracts. Both nonproteolytic acid proteins, involved in the degradation of several biological polymers, including membrane-associated protein, and small GTPases, involved in vesicular trafficking, were detected in each type of lysosome, even though liver lysosomes showed a protein % coverage lower than those identified in cells. In summary, these three biological matrices were similar concerning their protein lysosomal content. In virtue of this, HLLs can be considered a valid alternative to DC lysosomes for studying the Ag presentation and processing in APCs in vitro (Fig. 1).

**Table I.**
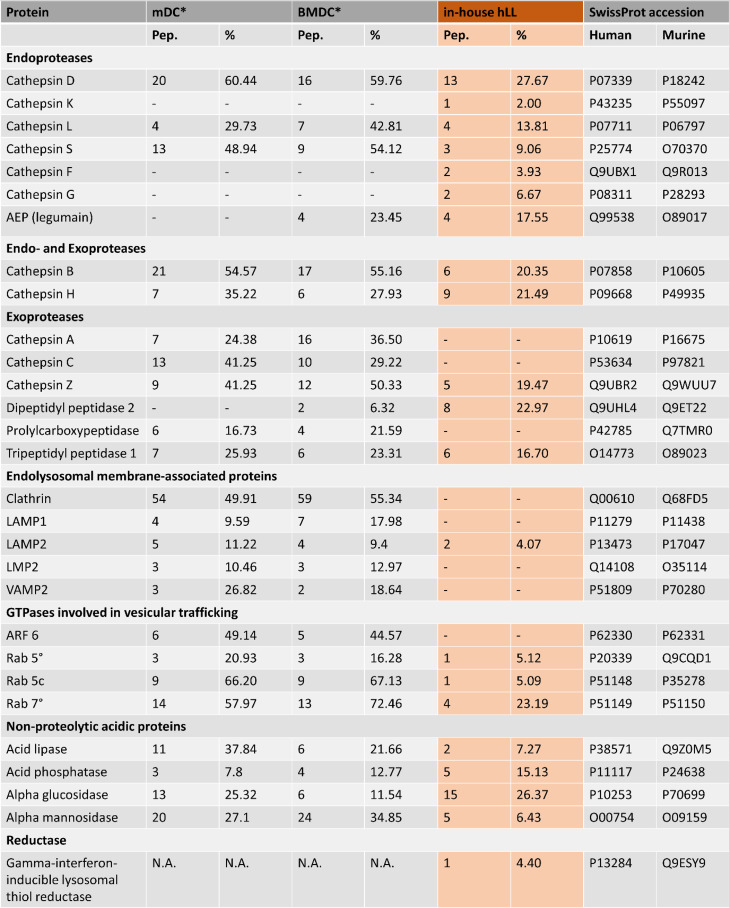
Comparison of the enzymatic lysosomal content among mDCs, BMDCs, and in-house hLLs

The number of identified peptides and the percentage to which identified peptides cover the full-length protein sequence are reported.

*Data from the MS-based fingerprinting of DC-derived lysosomal enzymes were taken from the literature (24).

AEP, asparagine endopeptidase; ARF, ADP-ribosylation factor; LAMP, lysosome-associated membrane glycoprotein; LMP, lysosome membrane protein; Pep., identified peptides; Rab, Ras-like protein; VAMP, vesicle-associated membrane protein.

### IFX lysosomal processing in several reducing conditions

**FIGURE 2.**
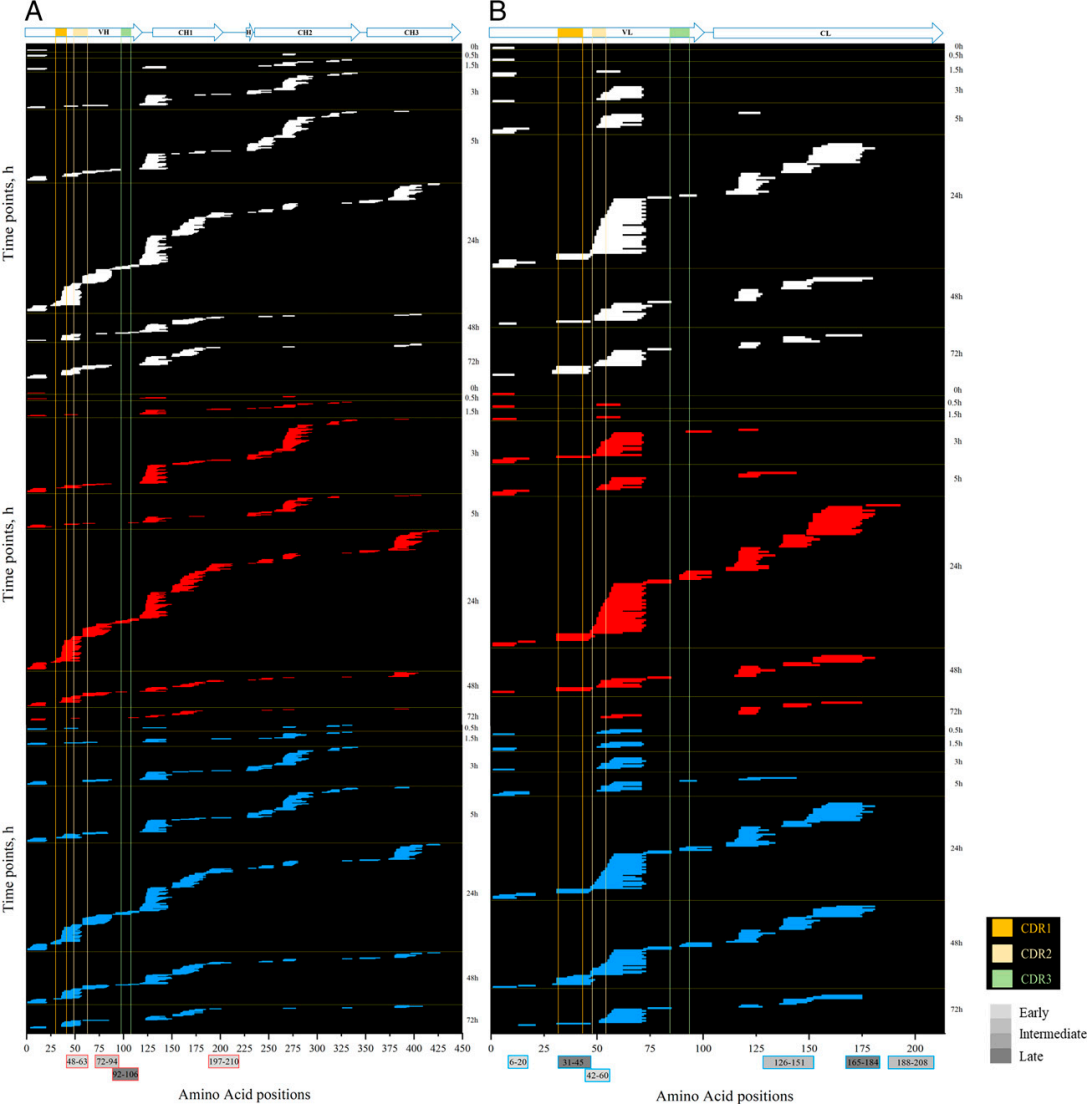
Chronology of IFX lysosomal degradation. Peptides were analyzed by high-resolution MS after 0, 0.5, 1.5, 3, 5, 24, 48, and 72 h of in vitro digestion with HLLs. (**A**) HC lysosomal degradation. (**B**) LC lysosomal degradation. Regions of predominant peptide clusters are shown as bars colored in different shades of gray according to their time occurrence (early appearance: ≤1.5 h, intermediate appearance: 1.5 h ≤ *t* < 5 h, late appearance: ≥5 h). Several redox conditions were considered: Cys 1 mM + DTT 2 mM (white), DTT 4 mM (red), and DTT 2 mM (blue). Peptide length considered ranged from 9 to 30 aa. CL, constant LC; H, hinge region; VH, variable HC; VL, variable LC.

**FIGURE 3.**
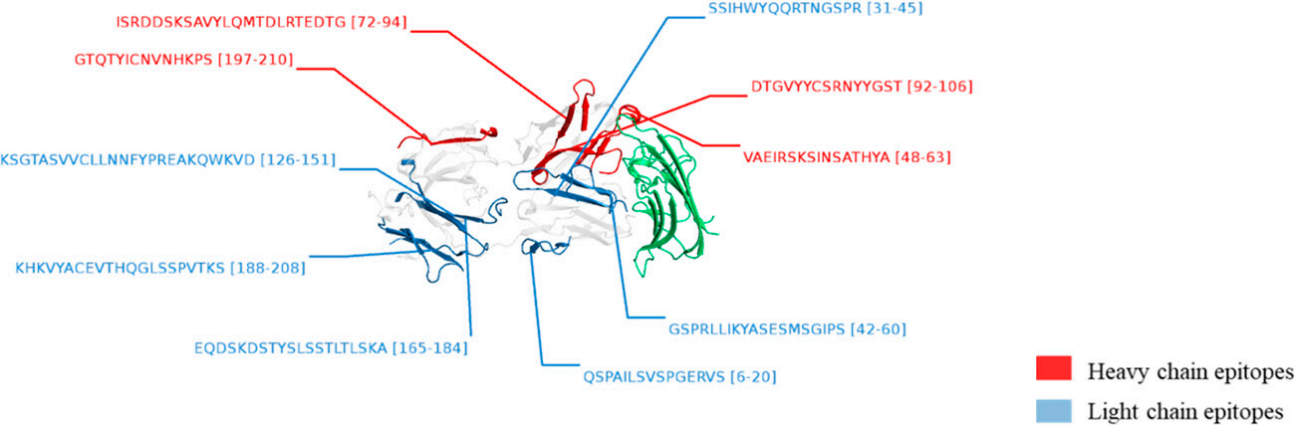
IFX peptide clusters exposed on the cell surface through MHCII molecules mapped onto a crystal structure of IFX Fab fragment in complex with target TNF-α (PDB ID: 4G3Y). These data were obtained by using an in-house–optimized MAPP method in which mDCs had been previously challenged with IFX for 24 h. Epitopes from HCs and LCs are in red and blue, respectively. Epitope lengths, shown in the figure, included all the peptide clusters located in that position, which shared a common central core. All the clusters identified by using MAPP assays were already formed in lysosomes in early time points.

We compared IFX lysosomal degradation in three different reducing conditions using HLLs as biological matrix. Peptides obtained by in vitro lysosomal degradation were then analyzed by exploiting HRAMS (Supplemental Fig. 1). After in vitro proteolysis, nested peptide clusters were formed with the same central cores and variable flanking regions. The variability of flanking regions was consistent with MHCII binding groove features, given that these binding pockets are characterized by open ends that allow peptides with different lengths, generally between 9 to 30 aa, to bind (Fig. 2). According to that, we considered only those peptides that fulfilled the requirements needed for MHCII binding during data analysis. Analyzing both the variable and constant chain, we observed that H chain (HC) and L chain (LC) hypervariable regions, CDRs CDR1 and CDR2, were abundantly cleaved during the lysosomal processing, although CDR3 appeared to be more protected against cathepsin proteolytic activity. Several peptide clusters of both CDR1 and CDR2 were already exhibited after 3 h from the beginning of IFX degradation, whereas CDR3 peptide clusters were visible only after 24 h. Given the number of formed peptides, we can assume that the cathepsin-cutting action is more effective on CDR1 and CDR2 with respect to CDR3 in different redox-tested conditions. Regarding C regions, we noted that most of the peptide clusters present after LC degradation were the same in all three redox conditions and were significantly abundant after 5 h, reaching the highest expression at 24 h. HC C region (CH) showed a similar degradation pattern in the three tested conditions. Fragments of CH1 region were detected even after half an hour, reaching the maximum number of clusters in 24 h. CH2 region showed similar degradation progress of CH1 even though cluster expression started after 90 min from the beginning of the reaction, reaching the top at 5 h, with a subsequent decline until 24 h. On the contrary, CH3 region peptides were significantly representative only after 24 h, given their absence in the previous time points. Regarding the C-terminal portion in both chains, the lack of potential cleavage sites targeted by proteolytic enzymes may explain the absence of peptide clusters identified in the LC C-terminal region. On the contrary, the C-terminal part of IFX HC appeared cleaved by proteases after 5 h of enzymatic digestion, even though the number of detected clusters was fewer than in other parts of the C region. Next, obtained results were compared with internal results derived from MAPP assays (Fig. 3) performed on the same IFX molecule (A. Di Ianni, T. Fraone, P. Balestra, K. Cowan, F. Riccardi Sirtori, and L. Barbero, submitted for publication). Most of the peptides and their related clusters detected on mDCs membranes were already formed before 24 h.

### IFX lysosomal degradation SDS-PAGE analysis confirmed MS analysis data

**FIGURE 4.**
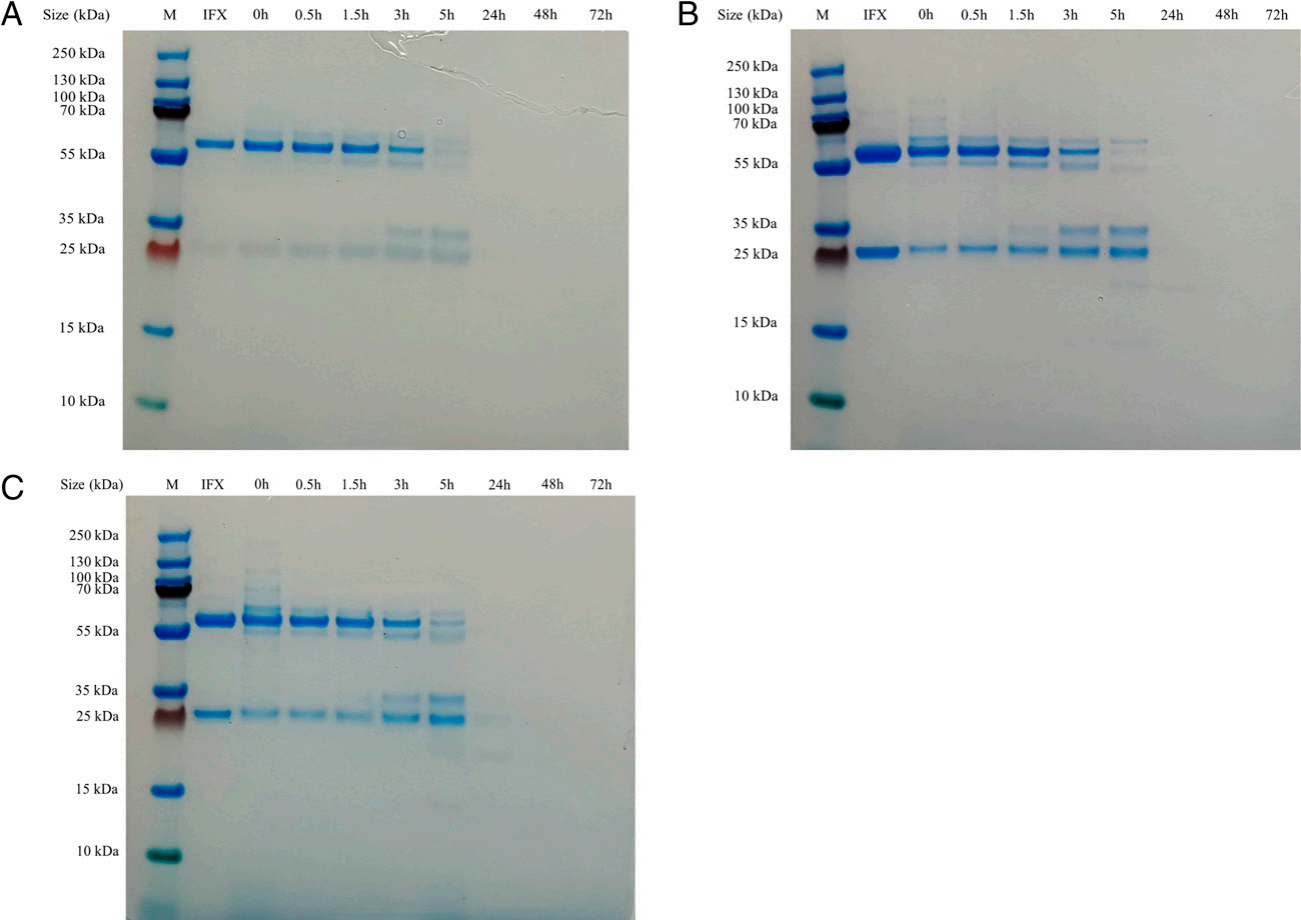
A total of 1.5 µg of protein samples was analyzed by SDS-PAGE and SimplyBlue SafeStain staining after 0, 0.5, 1.5, 3, 5, 24, 48, and 72 h of in vitro degradation using HLLs. A total of 4 µg of untreated protein was used as protein reference to track the degradation trend of both HCs and LCs. All three redox conditions were tested, and results were compared with those obtained using high-resolution MS (**A**, Cys 1 mM + DTT 2 mM; **B**, DTT 4 mM; **C**, DTT 2 mM).

To confirm the previously obtained data, we carried out SDS-PAGE separation to evaluate the degradation trend of the mAb over time (Fig. 4). IFX-treated HC bands showed a decreasing trend in terms of intensity, reaching, after 24 h, a concentration that was not detectable by the staining solution. IFX-treated LC bands also showed a declining trend in the first three time points, but their intensity appeared higher at 3–5 h because of the overlapping of LCs and portions of HCs with similar molecular weights. Portions of HC and LC fragments were visible below each respective band. In the last two lanes (48–72 h), there was no detectable peptide band.

### IFX degradation rate is slightly affected by redox microenvironment changes

**FIGURE 5.**
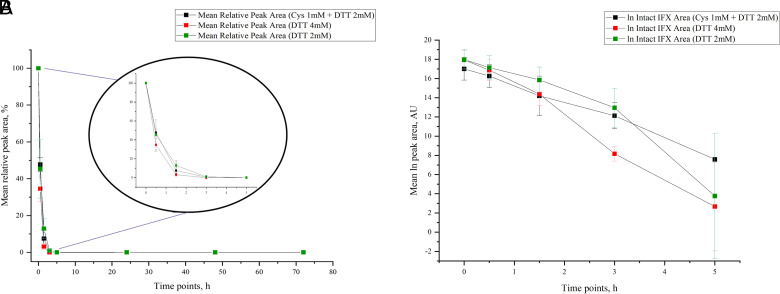
(**A**) IFX concentration decreased exponentially in time during the lysosomal processing. Peak area % related to *t* = 0 was plotted against time. No significant variances were visible in the three test conditions. In all three conditions, IFX was almost completely degraded after 2–3 h. The three conditions tested are depicted with different colors. Results for three independent measurements are shown; error bars show SD. (**B**) IFX degradation process can be summarized as a pseudo–first-order enzymatic kinetic by 3 h. Natural logarithm of the peak area is plotted against time. The slope of the linear functions depicted in the diagram represents the constant rate of the process, which is inversely proportional to the *t*_1/2_ of the reagent. Results for three independent measurements are shown.

The data obtained by LC-HRAMS analysis revealed that the lysosomal enzymatic activity on IFX, regardless of the redox condition applied, could be described by a pseudo–first-order kinetic, because the concentration of the mAb decayed over time according to an exponential function (Fig. 5A). This result was confirmed by the pseudolinear trend depicted in the semilogarithmic diagram below. IFX degradation appeared similar in HLLs spiked with 1 mM Cys in the presence of 2 mM DTT compared with the condition in which only 2 mM DTT was used as a reducing agent. In both cases, IFX disappeared after 5 h from the beginning of the incubation. The increase of the DTT amount (4 mM) in the biological matrix did not significantly increase the proteolysis rate. Overall, changing the redox lysosomal microenvironment did not significantly modify the mAb’s degradation rate, implying that all three trends were quite similar (Fig. 5B). To confirm this, we measured t_1/2_ values in these three conditions, obtaining the following results: 0.45 ± 0.04 h in the presence of Cys 1 mM and DTT 2 mM, 0.33 ± 0.06 h with DTT 4 mM, and 0.47 ± 0.18 h with DTT 2 mM (Table II).

**Table II.** Reported data represent the average values of three independent replicates for each experimental condition

	Best-Fit Values				SD Errors			Goodness of Fit				Constraints
	Span	*K*	Plateau	*t*_1/2_ (h)	Span	*K*	Plateau	Df	*R* ^2^	Absolute Sum of Squares	Sy.x	*K*
Cys 1 mM + DTT 2 mM	3.47E+07	1.54	−8.90E+04	0.451	3.68E+05	6.89E−02	1.57E+05	5.00	0.998	8.36E+11	3.47E+05	>0
DTT 2 mM	9.51E+07	1.66	1.09E+05	0.467	1.27E+06	7.74E−02	5.45E+05	5.00	0.999	1.12E+13	1.20E+06	>0
DTT 4 mM	8.55E+07	2.17	−2.39E+05	0.326	7.83E+05	2.94E−02	3.18E+05	5.00	1.000	6.96E+12	7.30E+05	>0

Data reveal a good correlation between the one-phase exponential regression curves and the trendlines. *t*_1/2_ are calculated in the three experimental conditions and shown below as *t*_1/2_ in hours. All data were calculated by GraphPad Prism 4.03.

Absolute sum of squares, values for coefficients to minimize the sum-of-squares of the differences between the predicted *y* values and the actual *y* values; Df, number of rows of data analyzed minus the number of parameters in the model; *K*, constant rate; plateau, *y* value at infinite times; *R*^2^, coefficient of determination; span, difference between *y*_0_ and plateau values; Sy.x, SD of residuals; *y*_0_, *y* value when *x* (time) is zero.

### Redox microenvironment changes did not alter the peptide fragments appearance rate

**FIGURE 6.**
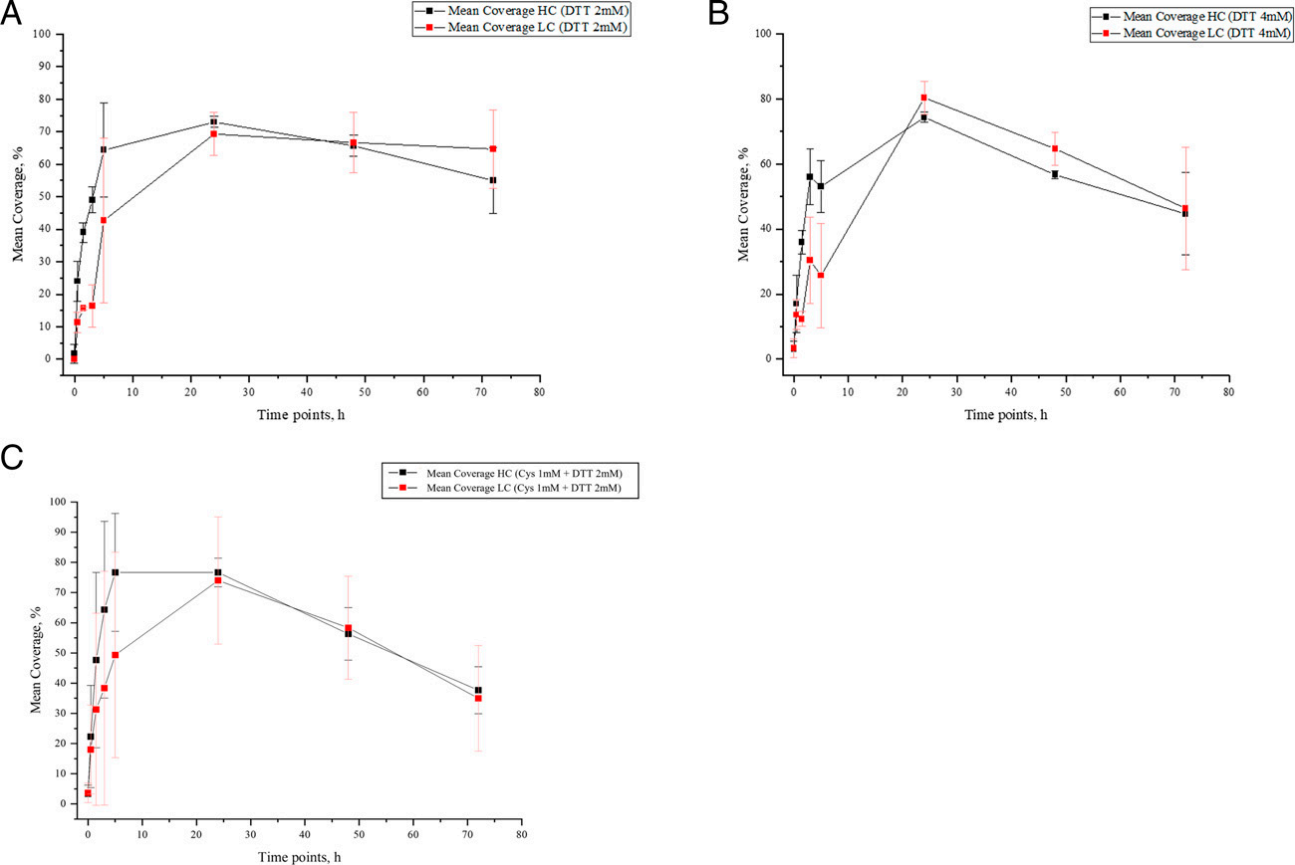
Peptide appearance speed was slightly affected by redox condition variation. (**A**–**C**) Line graphs and scatterplots describe the appearance of IFX HC and LC peptides in the presence of different reducing conditions. Percentage coverage score was taken as a parameter to follow proteolysis in lysosomes and evaluate its variation at each time point. Peptide length considered ranged from 9 to 30 aa. Results for three independent measurements are shown.

IFX enzymatic processing was analyzed starting from 0 h (first time point), when the mAb was still intact, and ending at 72 h (last time point), when both IFX HCs and LCs were metabolized entirely because of the cathepsin action. Before 3 h, the number of peptides generated from proteolysis in all three experimental conditions was still low, as reported by the coverage score. However, the lysosomal proteome quickly increased between 5 and 24 h, reaching a maximum value after 24 h for both the LCs and the HCs. In the last two time points (48 and 72 h), the coverage score decreased because of the action of cathepsins on small peptides. PD was set to detect only peptides with >8 aa. Therefore, the decrease in the coverage score may be explained by an increase of peptides no longer than 8 aa, which were undetectable. IFX spiked with 2 mM DTT exhibited a rapid degradation of both chains, reaching the top at 24 h (Fig. 6A). However, increasing the amount of DTT to 4 mM in the biological matrix did not improve the speed of peptide appearance given that the peak of coverage score was still reached at 24 h, as shown in Fig. 6B. As far as the presence of Cys in the matrix was concerned, the IFX processing trend seemed to be slightly affected. This was evident from a higher rate of HCs degradation peaking at 5 h. In contrast, the LC degradation rate reached the top at 24 h, as shown in Fig. 6C.

### Statistical analysis of biological replicates by LFQ

**FIGURE 7.**
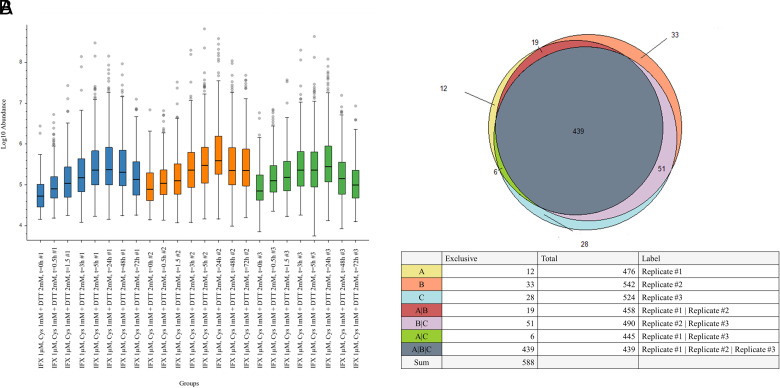
(**A**) LFQ analysis was performed by comparing each time point with the 24-h result. The sample abundances are shown as a box-and-whisker plot, which displays the peak intensity values for all the peptides detected. Data are shown in a semilogarithmic graph, where abundances values are expressed as decimal logarithm. Boxes represent the first and third quartiles of the dataset. Circles represent outlier data points. Group colors distinguish data from biological replicates (blue for replicate 1, orange for replicate 2, green for replicate 3). Error bars show SD. The bottom displays the increasing trend of peptide abundance during IFX lysosomal degradation in the presence of 1 mM Cys and 2 mM DTT. The other two tested conditions are shown in the Supplemental Fig. 2. (**B**) Peptides in common among replicates are shown by Euler-Venn graphs. Data displayed in the chart are related to the 24-h time point for each replicate considered. Letters are used to indicate which replicate is referred to, according to the following scheme: A, B, and C for the first, second, and third replicate, respectively. Exclusive peptides are shown in the table for each replicate or match of examined replicates. The chart below refers to IFX lysosomal degradation in the presence of 1 mM Cys and 2 mM DTT. The other two tested conditions are shown in the Supplemental Fig. 3.

An assessment of the robustness of this method concerning lysosomal degradation variability was conducted by comparing protein and peptide abundances with LFQ values under different redox conditions (Fig. 7A; Supplemental Fig. 2). This technique consists in quantifying proteins by integrating the peak intensity of each sample without pooling internal standards. For each redox condition, the 24-h time point was selected as a reference sample for quantification, because it was the one with the highest level of peptide abundance. Therefore, every time point was compared with the 24-h time point, and each abundance value was expressed as a decimal logarithm. Analyses were performed for all biological replicates (n = 3). Data revealed that, in terms of peptide abundances, a similar trend was observed in each experimental redox environment, reaching the highest number of peptides in the time frame between 24 and 72 h, where IFX was almost completely degraded. An evaluation of common peptides among the three replicates was performed to better define the degree of biological variability in replicates, considering 24 h as a reference. Overall, a similar behavior was shown in all three redox conditions, with many peptides identified in all three replicates. Besides, the number of exclusive peptides in each replicate was much lower than in the whole peptidome. These data implied that IFX lysosomal degradation was similar in each biological replicate for each experimented redox condition (Fig. 7B; Supplemental Fig. 3).

## Discussion

In recent years, biotherapeutics have provided valuable medicine to fight diseases that once were not pharmacologically treatable and were responsible for the deaths of many patients. The growing request for novel therapies for treating these diseases has led to increasing the development, production, and market authorization for new therapeutic biomolecules. However, several challenges must be considered when it comes to therapeutic protein development, especially regarding the evaluation of their safety and efficacy on patients. The accurate assessment of the biotherapeutic immunogenicity still represents a great challenge for scientists because a multitude of endogenous components play a key role in eliciting immune responses against therapeutic proteins, and in many cases, a combination of more factors is involved in the development of specific adverse events. In the last few years, there has been a rapid development of novel in vitro systems capable of predicting and assessing drug immunogenicity in the early stages of development (39–41). Unfortunately, these predicting tools require numerous data to generate satisfactory and reliable results considering the high complexity of immune responses. Hence multiple approaches need to be considered to succeed in this goal. In this study, we described the development of an LC-HRAMS method that combines the evaluation of catabolic products of biologicals to their degradation rate as intact species as an alternative approach to studying biologics proteolysis instead of using more consolidated biochemical techniques. In this work, SDS-PAGE was performed only as an orthogonal technique to confirm data from MS. We examined lysosome degradation kinetics because Ag processing plays a pivotal role in immunogenicity. We also focused on the activity of the only reductase present in the lysosomal compartments, GILT (42, 43). During the entire chromatography run, the reported chromatography and MS parameters were set to allow complete identification of both intact proteins and their related peptides. High resolution was reached by exploiting nanoflow rates on UHPLC systems, even though the chromatography run time was strongly impacted. However, using a combination of two trapping columns with different stationary phases allowed us to get a remarkable improvement in terms of peptide separation efficiency, detection, and resolution. In vitro Ag processing was performed using a surrogate model of human APC lysosomes. Because cost, time, up-scaling ability, and standardization problems make it difficult to get lysosomes from human APCs, using a surrogate model might be a good solution. The qualitative comparison of the lysosomal contents of the human liver with literature data for mDCs and BMDCs (24) showed that they all contained similar enzymatic content. An MS bottom-up proteomic approach was used for this purpose, exploiting nonmagnetic trypsin-coated beads in a proper digestion buffer to cleave the biological matrix’s enzymatic fraction. In doing so, we observed that most of the crucial lysosomal enzymes in DCs involved in the MHCII pathway processing were also present in other cells. Thus, it is conceivable that hLL might be a substitute in in vitro models to evaluate Ag processing in professional APCs. Due to its ability to activate the immune system and generate ADAs, IFX was chosen as a test item in degradation assays. Parallel mAb processing experiments have been conducted in the presence of several reducing agents to assess the influence of several redox environments on GILT activity. DTT was added to each solution as a reducing agent to restore reduced GILT and allow full catabolic activation of Cys cathepsins. Moreover, we also examined the impact of redox changes on proteolytic enzyme activity by adding a supplemental reducing agent to a fixed DTT concentration (2 mM). Three different patterns of lysosomal degradation kinetics were observed. In the case of the sample treated with 2 and 4 mM DTT, the peak of cleavage and formation of smaller peptides from both LCs and HCs occurred at 24 h. On the contrary, samples containing both reducing agents showed a different trend because LC and HC maximum degradation was reached at different time points. The IFX degradation kinetics could be accurately described by an exponential decay function, which confirms the pseudo–first-order kinetics of the lysosomal enzymatic activity. Combining the reducing agents (Cys and DTT) resulted in a similar lysosomal activity on the test item compared with the other tested conditions. Samples treated with only DTT did not show significant differences in proteolytic activity and peptide appearance (Figs. 5, 6). In other words, no significant impact in proteolysis was detected between the three conditions, suggesting that MHCII peptide complexes are usually presented to CD4+ Th cells between 5 and 24 h. These findings were also in line with t_1/2_ calculated in the three different reducing conditions, indicating that changing the lysosomal redox environment did not affect the stability of the mAb in the lysosomal fraction. Comparing MAPP results of IFX-exposed mDCs with the peptides pool obtained after proteolysis, we also noticed that many of the detected clusters were already present in the time frame between 3 and 24 h. Moreover, T-cell activation assays demonstrated that those epitopes contributed to eliciting a CD4+ T cell response in 12 of 15 healthy donors, whereas patients who had previously received IFX treatment and developed dose-limiting levels of ADAs showed a positive response to at least one of those epitopes (44). Homann et al. (45) also described that four peptide clusters in the variable HCs and LCs and two peptide clusters in the constant HCs were recognized not only by ADAs in sera of 20 IFX-treated patients, but also in healthy control donors, confirming the presence of pre-existing ADAs even in an untreated population. However, it is important to underline that these degradation processes might be quicker in vivo, because proteolytic enzymes in intact lysosomes are more concentrated. Nevertheless, the assay can be a useful tool for assessing the course of in vivo processing, given the presence of the full proteolytic machinery in the lysosomal extracts we used. In conclusion, we developed an LC-HRAMS approach for optimizing MAPP assays, especially considering that the incubation step of proteins with immature DCs and their following maturation is time consuming. Our findings show that biotherapeutics proteolysis in the MHCII pathway is optimal in the time frame between 5 and 24 h, setting an important parameter for the MAPP method development. In this context, HLLs from a mixed gender pool of four donors revealed themselves as a good in vitro model system to mimic the MHCII compartments of APCs and consequently to study the Ag proteolysis. To validate this method, it will be required to analyze the processing of different biological drugs and to expand the lysosomal pool, including a significant number of donors from the general population.

## Supplementary Material

Supplemental Figures 1 (PDF)
