# Supplementary material for: Development of a Liquid Chromatography and High-Resolution and -Accuracy Mass Spectrometry Method to Evaluate New Biotherapeutic Entity Processing in Human Liver Lysosomes
Source: Immunohorizons. 2023 Jun 16;7(6):467–79. doi: 10.4049/immunohorizons.2300035 (PMC10580112; doi:10.4049/immunohorizons.2300035)
Supplement: Supplemental Figures 1 (PDF) [file IH_2300035_Supplemental_1.pdf]

LC-HRAMS method for biotherapeutic processing in lysosomes

A

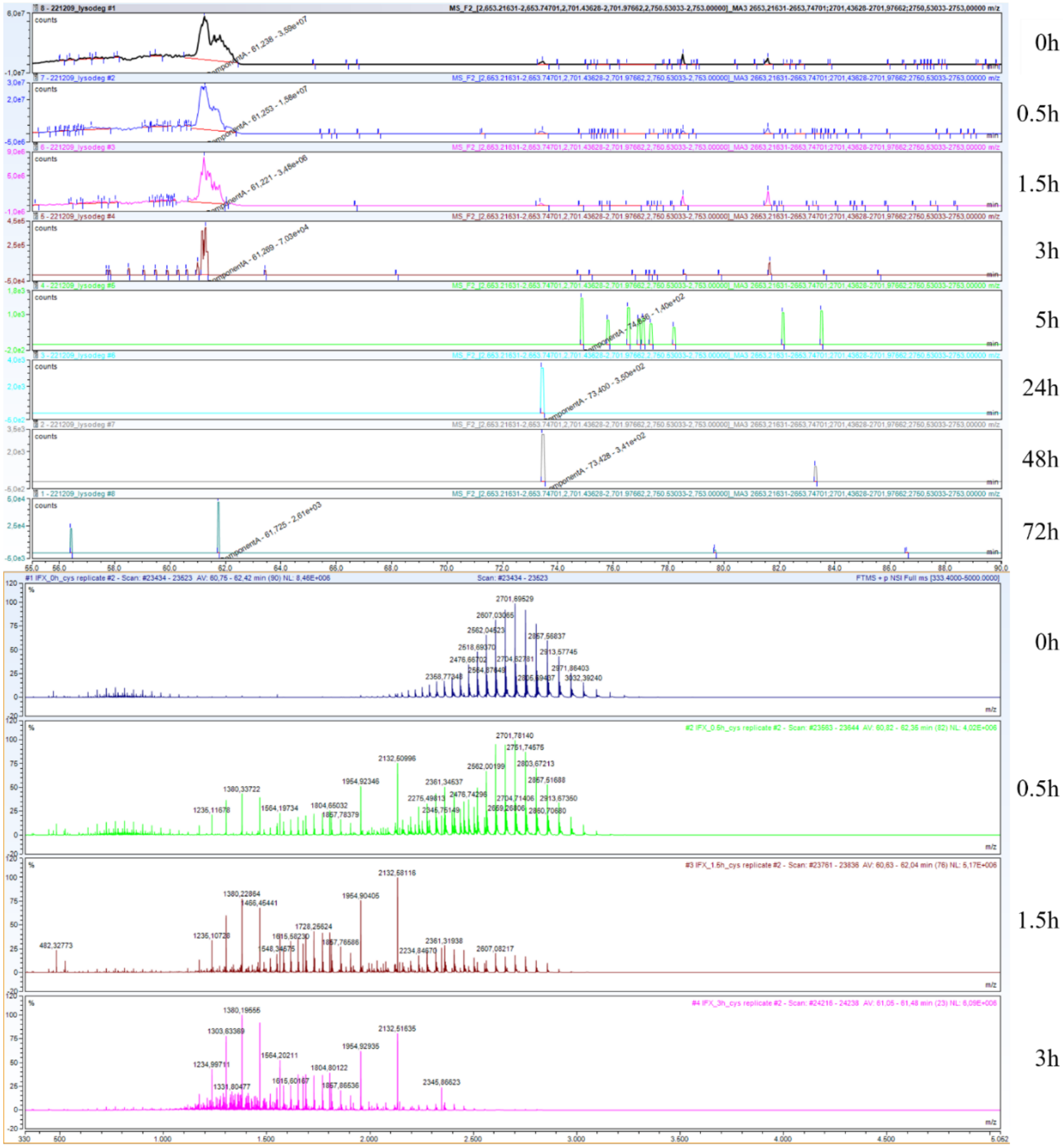

B

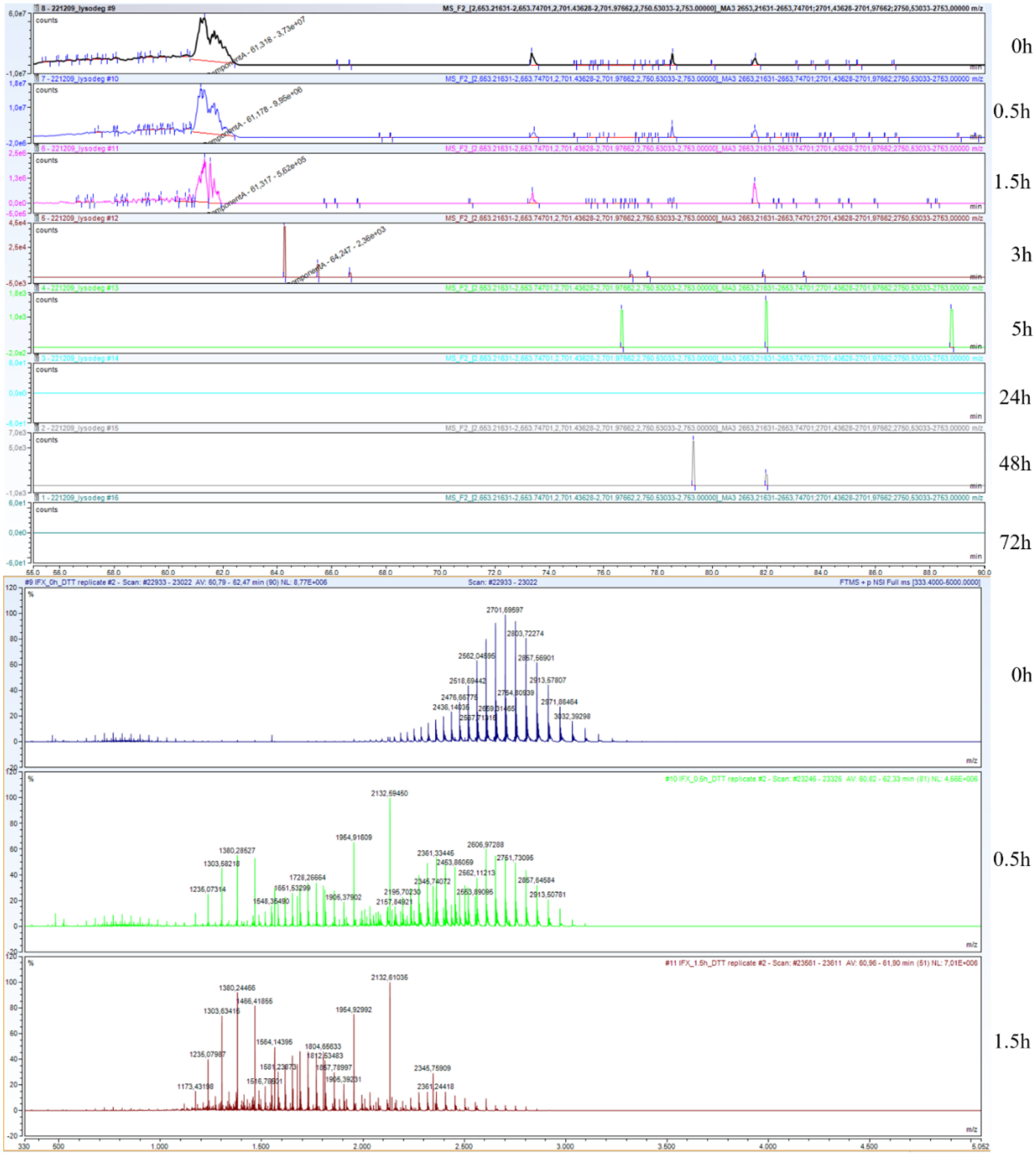

C

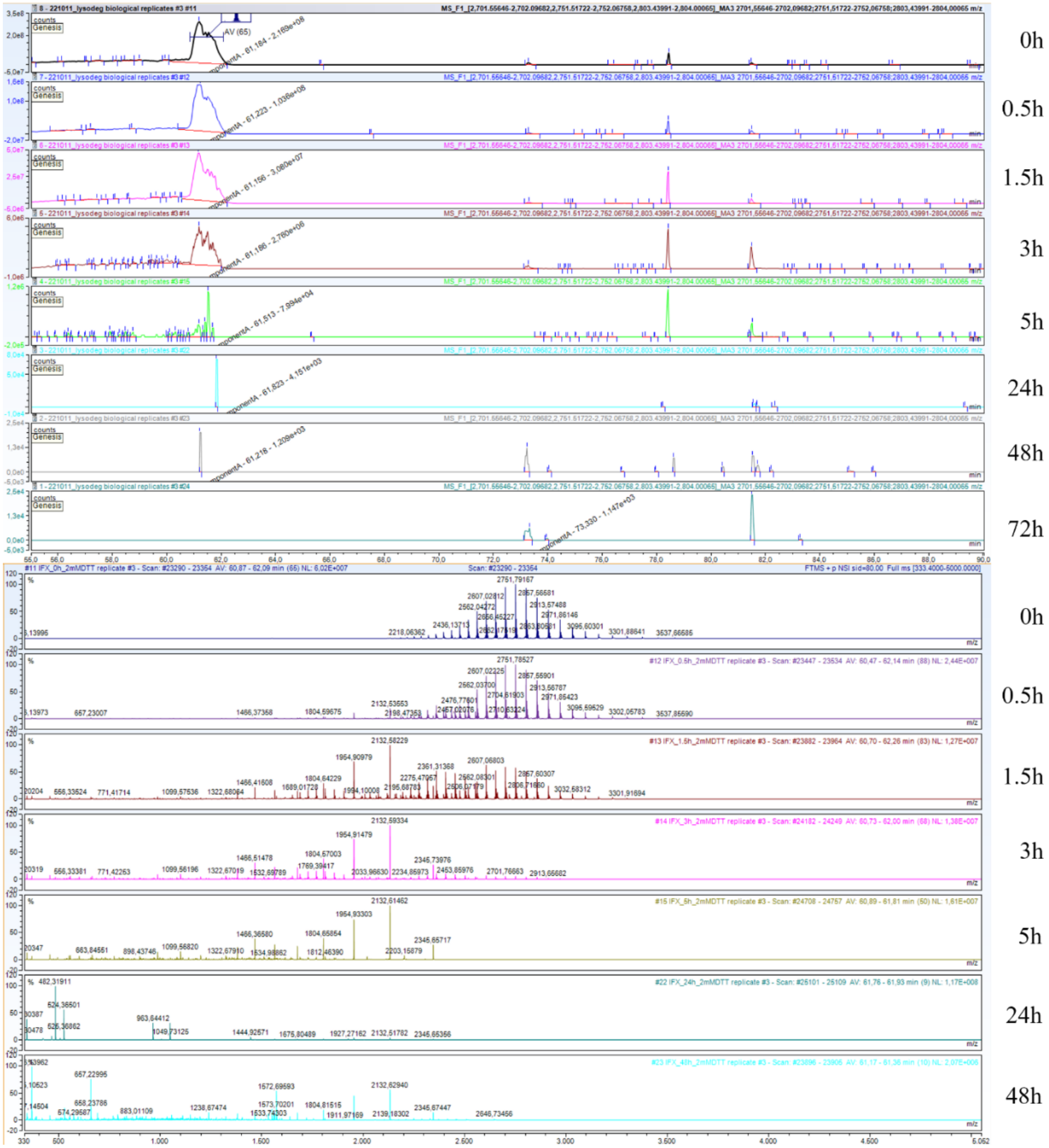

FIGURE S1 | Extracted ion chromatograms (XICs) and related mass spectra of IFX degradation in presence of (**A**) Cys 1mM and DTT 2mM, (**B**) DTT 4mM, (**C**) DTT 2mM as reducing agents. Time points from 0 to 72 h are shown in the figures. Retention time is around 61±0.5 min, according to the features of the applied method. Chromatograms highlight the decrease of the peak area vs time as long as intact antibody is still detectable. Mass spectra display the same trend, shedding a light on the separation of heavy and light chains and the formation of big, chunked peptides, which turn into smaller ones until their complete disappearance. IFX isotopic distribution is shown at 0 h, whilst heavy and light chain degradation becomes evident after 1.5 h. At 72 h, full degradation is finally reached. Chromeleon™ 7.2.10 Chromatography Data System (CDS) software (ThermoFisher™ Scientific) was used to obtain both chromatographic and MS data.

A

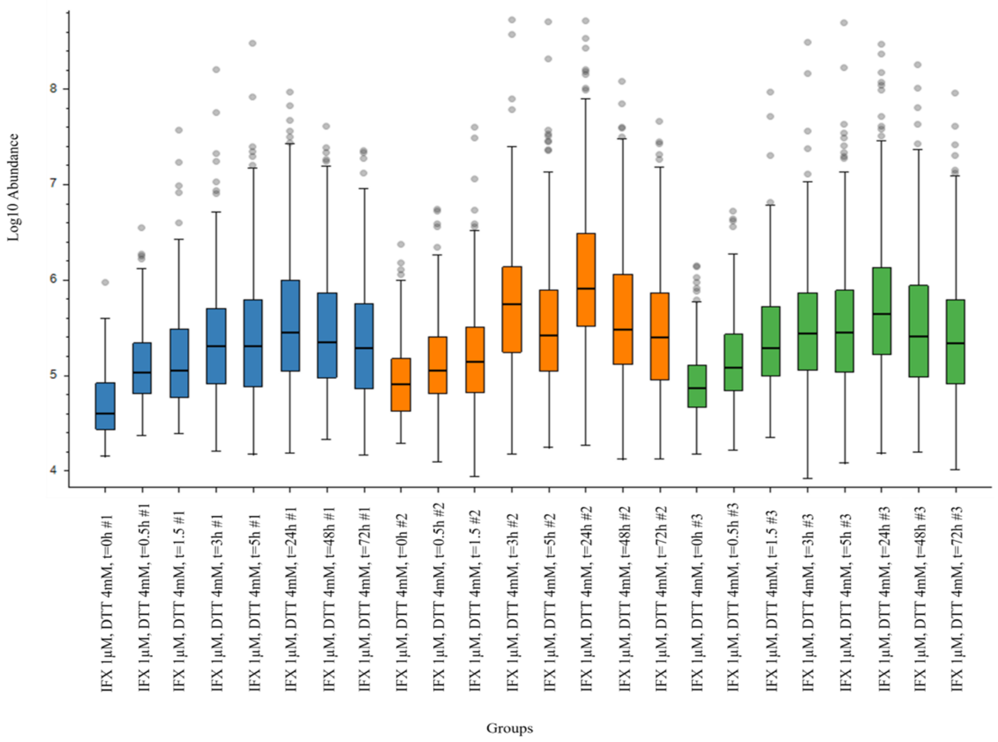
Log10 Abundance
4
5
6
7
8
IFX 1µM, DTT 4mM, t=0h #1
IFX 1µM, DTT 4mM, t=0.5h #1
IFX 1µM, DTT 4mM, t=1.5 #1
IFX 1µM, DTT 4mM, t=3h #1
IFX 1µM, DTT 4mM, t=5h #1
IFX 1µM, DTT 4mM, t=24h #1
IFX 1µM, DTT 4mM, t=48h #1
IFX 1µM, DTT 4mM, t=72h #1
IFX 1µM, DTT 4mM, t=0h #2
IFX 1µM, DTT 4mM, t=0.5h #2
IFX 1µM, DTT 4mM, t=1.5 #2
IFX 1µM, DTT 4mM, t=3h #2
IFX 1µM, DTT 4mM, t=5h #2
IFX 1µM, DTT 4mM, t=24h #2
IFX 1µM, DTT 4mM, t=48h #2
IFX 1µM, DTT 4mM, t=72h #2
IFX 1µM, DTT 4mM, t=0h #3
IFX 1µM, DTT 4mM, t=0.5h #3
IFX 1µM, DTT 4mM, t=1.5 #3
IFX 1µM, DTT 4mM, t=3h #3
IFX 1µM, DTT 4mM, t=5h #3
IFX 1µM, DTT 4mM, t=24h #3
IFX 1µM, DTT 4mM, t=48h #3
IFX 1µM, DTT 4mM, t=72h #3
Groups

B

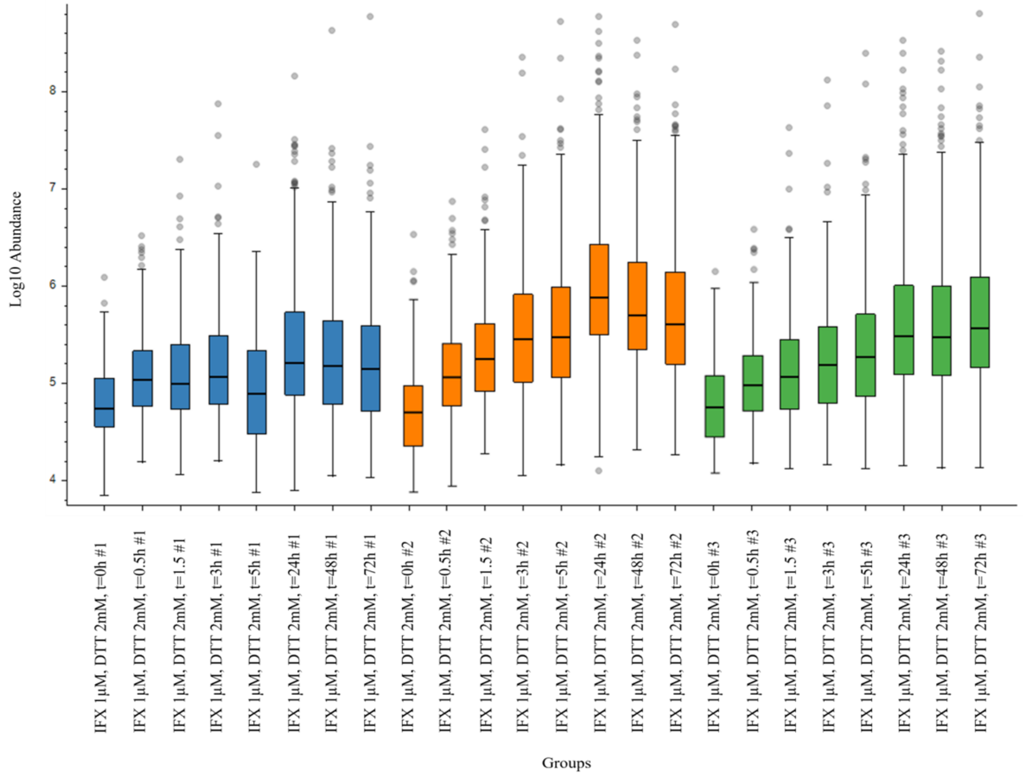
Log10 Abundance
4
5
6
7
8
IFX 1µM, DTT 2mM, t=0h #1
IFX 1µM, DTT 2mM, t=0.5h #1
IFX 1µM, DTT 2mM, t=1.5 #1
IFX 1µM, DTT 2mM, t=3h #1
IFX 1µM, DTT 2mM, t=5h #1
IFX 1µM, DTT 2mM, t=24h #1
IFX 1µM, DTT 2mM, t=48h #1
IFX 1µM, DTT 2mM, t=72h #1
IFX 1µM, DTT 2mM, t=0h #2
IFX 1µM, DTT 2mM, t=0.5h #2
IFX 1µM, DTT 2mM, t=1.5 #2
IFX 1µM, DTT 2mM, t=3h #2
IFX 1µM, DTT 2mM, t=5h #2
IFX 1µM, DTT 2mM, t=24h #2
IFX 1µM, DTT 2mM, t=48h #2
IFX 1µM, DTT 2mM, t=72h #2
IFX 1µM, DTT 2mM, t=0h #3
IFX 1µM, DTT 2mM, t=0.5h #3
IFX 1µM, DTT 2mM, t=1.5 #3
IFX 1µM, DTT 2mM, t=3h #3
IFX 1µM, DTT 2mM, t=5h #3
IFX 1µM, DTT 2mM, t=24h #3
IFX 1µM, DTT 2mM, t=48h #3
IFX 1µM, DTT 2mM, t=72h #3
Groups

C

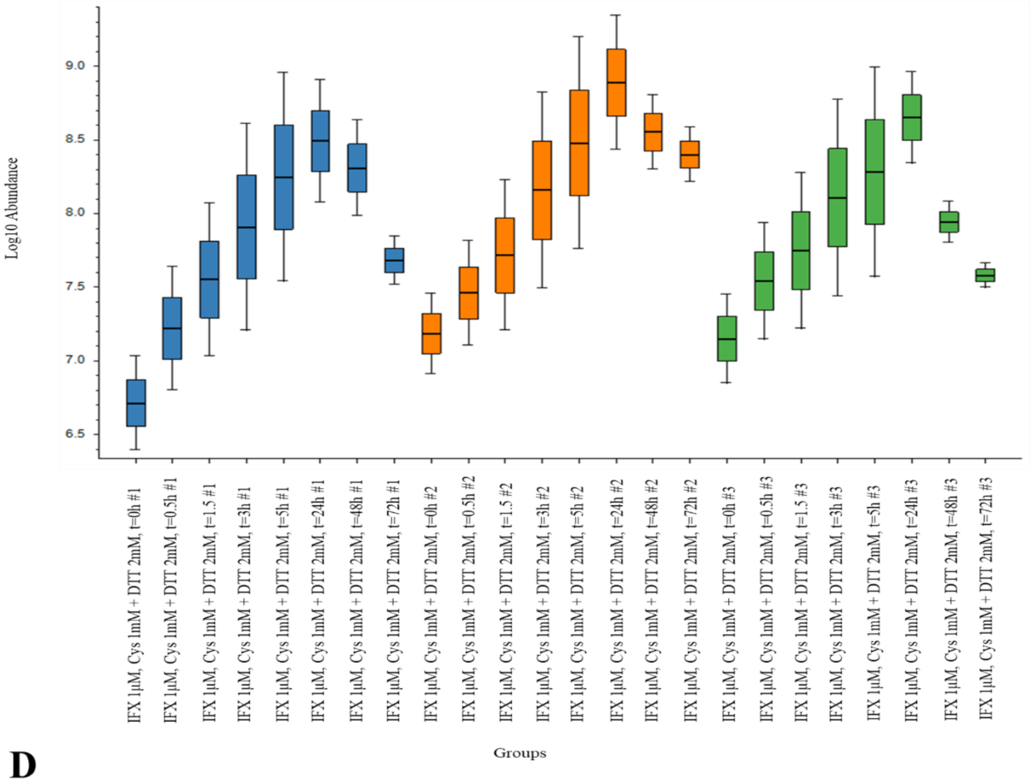
Log10 Abundance
6.5
7.0
7.5
8.0
8.5
9.0
Groups
IFX 1µM, Cys 1mM + DTT 2mM, t=0h #1
IFX 1µM, Cys 1mM + DTT 2mM, t=0.5h #1
IFX 1µM, Cys 1mM + DTT 2mM, t=1.5 #1
IFX 1µM, Cys 1mM + DTT 2mM, t=3h #1
IFX 1µM, Cys 1mM + DTT 2mM, t=5h #1
IFX 1µM, Cys 1mM + DTT 2mM, t=24h #1
IFX 1µM, Cys 1mM + DTT 2mM, t=48h #1
IFX 1µM, Cys 1mM + DTT 2mM, t=72h #1
IFX 1µM, Cys 1mM + DTT 2mM, t=0h #2
IFX 1µM, Cys 1mM + DTT 2mM, t=0.5h #2
IFX 1µM, Cys 1mM + DTT 2mM, t=1.5 #2
IFX 1µM, Cys 1mM + DTT 2mM, t=3h #2
IFX 1µM, Cys 1mM + DTT 2mM, t=5h #2
IFX 1µM, Cys 1mM + DTT 2mM, t=24h #2
IFX 1µM, Cys 1mM + DTT 2mM, t=48h #2
IFX 1µM, Cys 1mM + DTT 2mM, t=72h #2
IFX 1µM, Cys 1mM + DTT 2mM, t=0h #3
IFX 1µM, Cys 1mM + DTT 2mM, t=0.5h #3
IFX 1µM, Cys 1mM + DTT 2mM, t=1.5 #3
IFX 1µM, Cys 1mM + DTT 2mM, t=3h #3
IFX 1µM, Cys 1mM + DTT 2mM, t=5h #3
IFX 1µM, Cys 1mM + DTT 2mM, t=24h #3
IFX 1µM, Cys 1mM + DTT 2mM, t=48h #3
IFX 1µM, Cys 1mM + DTT 2mM, t=72h #3

D

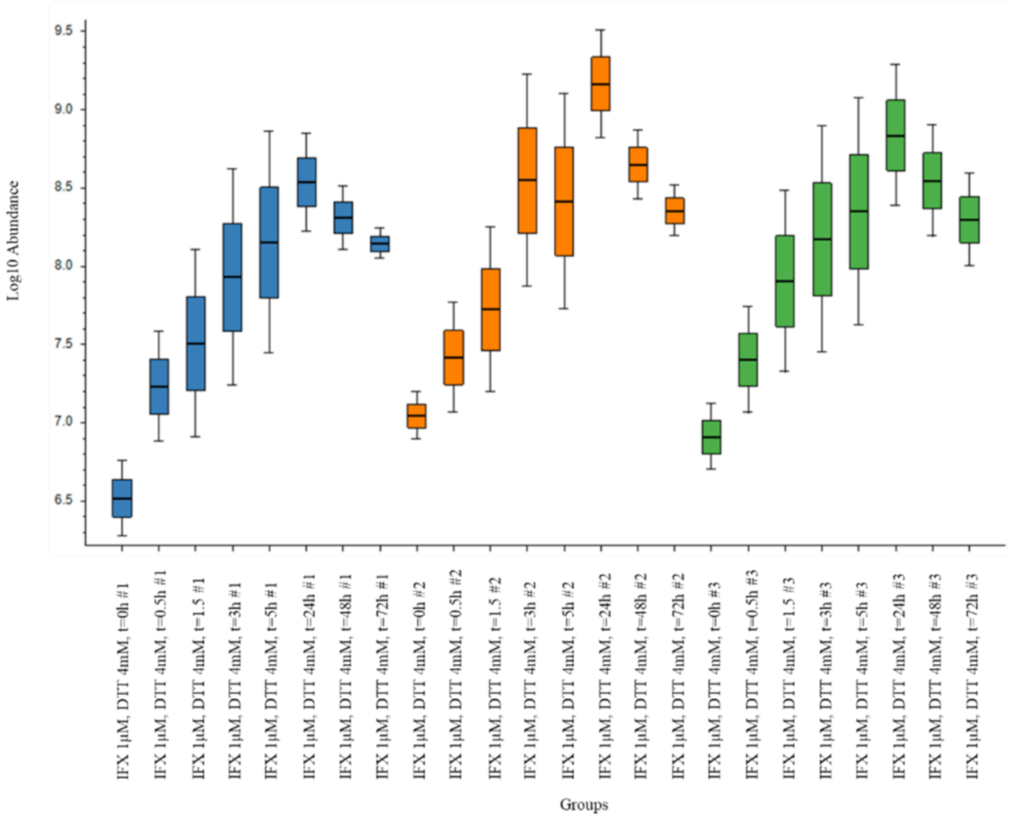
Log10 Abundance
6.5
7.0
7.5
8.0
8.5
9.0
9.5
Groups
IFX 1µM, DTT 4mM, t=0h #1
IFX 1µM, DTT 4mM, t=0.5h #1
IFX 1µM, DTT 4mM, t=1.5 #1
IFX 1µM, DTT 4mM, t=3h #1
IFX 1µM, DTT 4mM, t=5h #1
IFX 1µM, DTT 4mM, t=24h #1
IFX 1µM, DTT 4mM, t=48h #1
IFX 1µM, DTT 4mM, t=72h #1
IFX 1µM, DTT 4mM, t=0h #2
IFX 1µM, DTT 4mM, t=0.5h #2
IFX 1µM, DTT 4mM, t=1.5 #2
IFX 1µM, DTT 4mM, t=3h #2
IFX 1µM, DTT 4mM, t=5h #2
IFX 1µM, DTT 4mM, t=24h #2
IFX 1µM, DTT 4mM, t=48h #2
IFX 1µM, DTT 4mM, t=72h #2
IFX 1µM, DTT 4mM, t=0h #3
IFX 1µM, DTT 4mM, t=0.5h #3
IFX 1µM, DTT 4mM, t=1.5 #3
IFX 1µM, DTT 4mM, t=3h #3
IFX 1µM, DTT 4mM, t=5h #3
IFX 1µM, DTT 4mM, t=24h #3
IFX 1µM, DTT 4mM, t=48h #3
IFX 1µM, DTT 4mM, t=72h #3

E

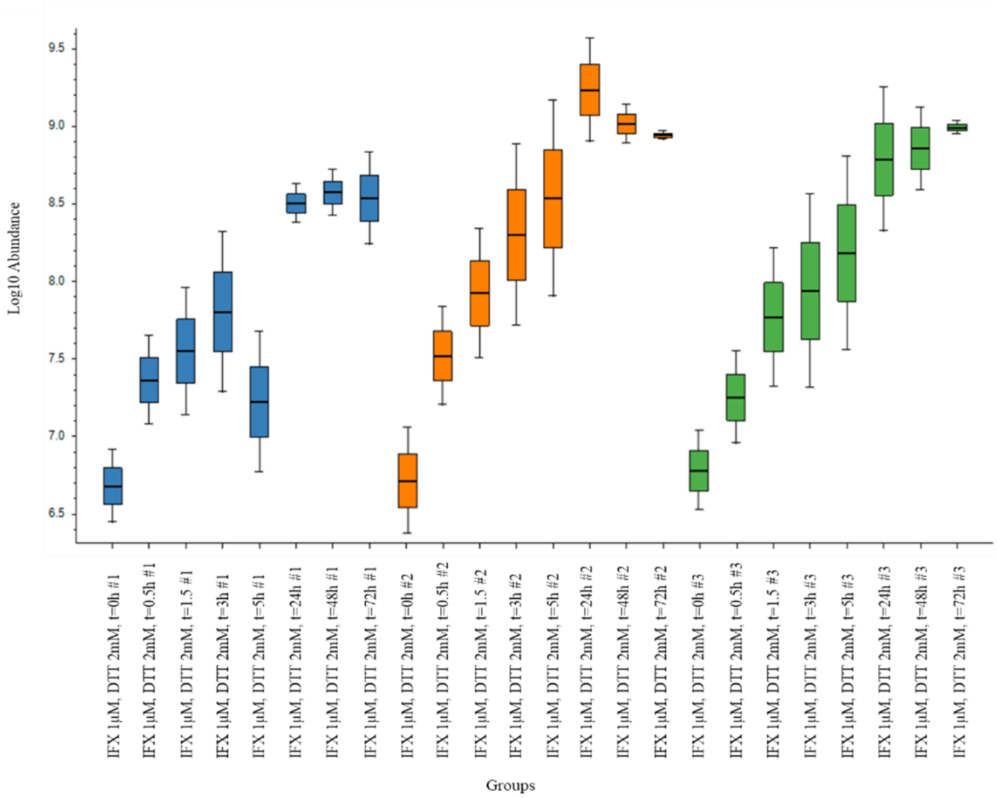

FIGURE S2 │ 1. Peptide abundance during IFX lysosomal degradation in presence of (**A**) 4mM DTT, (**B**) 2mM DTT. Data are shown as a semi-logarithmic box-and-whisker plot, where abundances values are expressed as decimal logarithm. Boxes represent the first and third quartiles of the data set. Circles represent outlier data points. Group colors distinguish data from biological replicates (blue for replicate #1, orange for replicate #2, green for replicate #3). Error bars show as standard deviation. 2. **C-E.** Protein group abundances for each time point considered are shown in the following charts. Box-and-whisker plots display the sample abundances for all the proteins detected in every performed biological replicate. Data are shown in a semi-logarithmic graph. Boxes represent the first and third quartiles for the data set. Biological replicates are depicted in three different colors. Results for independent measurements (n=3) are shown; error bars show standard deviation. Chromeleon™ 7.2.10 Chromatography Data System (CDS) software (ThermoFisher™ Scientific) was used to analyze mass spectrometry raw data. An overall increasing trend of protein group abundance is displayed for each redox tested condition.

I

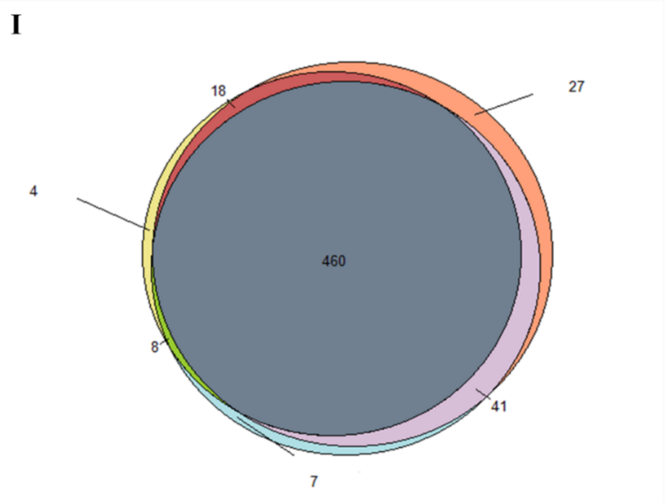

	Exclusive	Total	Label
A	4	490	Replicate #1
B	27	546	Replicate #2
C	7	516	Replicate #3
A|B	18	478	Replicate #1 | Replicate #2
B|C	41	501	Replicate #2 | Replicate #3
A|C	8	468	Replicate #1 | Replicate #3
A|B|C	460	460	Replicate #1 | Replicate #2 | Replicate #3
Sum	565		

II

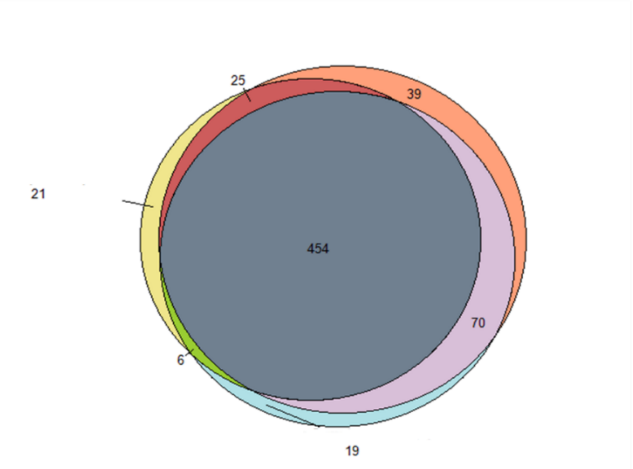

	Exclusive	Total	Label
A	21	506	Replicate #1
B	39	588	Replicate #2
C	19	549	Replicate #3
A|B	25	479	Replicate #1 | Replicate #2
B|C	70	524	Replicate #2 | Replicate #3
A|C	6	460	Replicate #1 | Replicate #3
A|B|C	454	454	Replicate #1 | Replicate #2 | Replicate #3
Sum	634		

FIGURE S3 | Peptides in common among replicates are shown by Eulero-Venn graphs. Data displayed in the chart are related to 24 h time point for each replicate and condition considered (in presence of 4mM DTT (**I**), 2mM DTT (**II)**). Letters are used to indicate which replicate is referred to, according to the following scheme: A, B, C for the first, second and third replicate, respectively. F number is related to sample group generated after uploading raw data file in Proteome Discoverer. Exclusive peptides are shown in the table for each replicate or match of replicates examined.
